# Single‐nucleus RNA sequencing reveals hippocampal cell type‐specific transcriptional alterations in Alzheimer’s‐linked *ACE* variant R1284Q knock‐in mice

**DOI:** 10.1002/alz.093021

**Published:** 2025-01-03

**Authors:** Miranda A Salvo, Leah K Cuddy, Sidhanth Chandra, Abhirami Ramakrishnan, Natalie Piehl, Thomas J Watson, David Gate, Jelena Popovic, Dmitry Prokopenko, Rudolph E. Tanzi, Robert J. Vassar

**Affiliations:** ^1^ Northwestern University, Chicago, IL USA; ^2^ Massachusetts General Hospital, Charlestown, MA USA; ^3^ Feinberg School of Medicine, Northwestern University, Chicago, IL USA

## Abstract

**Background:**

Angiotensin‐converting enzyme (*ACE*) is a validated risk locus for developing late‐onset Alzheimer’s disease (LOAD). ACE1 controls blood pressure through the renin‐angiotensin system (RAS), but it is also present and acts locally in the brain. Hypertension is associated with an increased risk for developing AD, and people taking select RAS‐targeting therapeutics have reduced incidence of AD. The *ACE* variant rs4980 (R1284Q murine mutation) was discovered in LOAD families through WGS. Our group previously showed that ACE1 R1284Q caused age‐associated hippocampal neurodegeneration and gliosis in mutant knock‐in (KI) mice, which was more aggressive in females. Importantly, these phenotypes were rescued by treatment with anti‐hypertensive drugs. Our previous study showed that ACE1 R1284Q caused neuron death in mice, however the mechanism is still unknown. This work aims to identify vulnerable hippocampal cell populations and pathways which might clarify the mechanism of ACE1 R1284Q‐mediated neurodegeneration.

**Method:**

Single nuclei were extracted from 30mg of flash‐frozen hippocampi from 6‐, and 12‐month‐old R1284Q ACE^+/+^ (WT) and ACE^KI/KI^ (KI) and processed using 10X Genomics 3’ Dual‐Index chemistry. Libraries were sequenced using the NovaSeq 6000. Preprocessing, quality control, and integration was performed on reads before subjecting them to cluster, differential expression, and pathway analyses.

**Result:**

Together, the samples were represented by 143,000 nuclei in 23 clusters encompassing all neuronal and glial populations in the hippocampus. We identified the expression of every RAS component, excluding renin, in the hippocampus. Notably, inhibitory and excitatory neurons had the most differentially expressed genes (DEGs) in 12‐month KI samples compared to controls. Additionally, 12‐month KI females had upregulated microglial C1q genes compared to males.

**Conclusion:**

We found expression of RAS genes in the hippocampus, which to our knowledge has not yet been characterized by single‐nucleus RNA sequencing. Furthermore, inhibitory neuron transcriptomes are the most affected in 12‐month KI mice, suggesting altered neuron communication that may lead to neuron loss. Additionally, we identified sex‐specific differences in 12‐month KI female microglia, which may explain the more aggressive gliosis seen in female KIs compared to males. Future directions include performing ingenuity pathway analysis to identify upstream regulators of DEGs and experimental perturbations to test our hypotheses.

